# Medication therapy management in Pakistan: a cross-sectional evaluation of pharmacists’ knowledge, attitudes, practices, and barriers

**DOI:** 10.1186/s40780-025-00493-8

**Published:** 2025-10-06

**Authors:** Muhammad Amir Hamza, Faiqa Imran, Aayza Ahmed, Wajeeha Ali, Hamza Siddique, Hina Ahsan, Babar Murtaza, Zaheer-Ud-Din Babar, Ali Ahmed

**Affiliations:** 1 Department of Pharmacy Practice, Faculty of Pharmaceutical Sciences, Riphah Institute of Pharmaceutical Sciences, Riphah International University, Islamabad, Pakistan; 2 Department of Pharmacy Practice, Faculty of Pharmaceutical Sciences, Hamdard Institute of Pharmaceutical Sciences, Hamdard University, Islamabad, Pakistan; 3 Department of Clinical Pharmacy and Practice, College of Pharmacy, QU Health, Qatar University, Doha, Qatar; 4 Division of Infectious Diseases and Global Public Health, School of Medicine, University of California, San Diego (UCSD), La Jolla, San Diego, CA USA

**Keywords:** Medication therapy adherence, Pharmaceutical services, Pharmacy practice, Drug therapy management, Patient-centered care, Health outcomes

## Abstract

**Background:**

Medication therapy management (MTM) is a patient-centered clinical service designed to optimize therapy and enhance patients’ quality of life. The published research has shown that pharmacists’ MTM services improve health outcomes. Collaboration among pharmacists, patients, and other healthcare providers is essential to ensure the safe and effective use of medicines. Despite its benefits, countries like Pakistan still face challenges in raising awareness of and implementing MTM. This study aimed to assess the current knowledge, attitudes, and practices of community and hospital pharmacists regarding MTM and identify the barriers to their implementation.

**Methods:**

An observational cross-sectional study was conducted between May 2024 and August 2024 using a pre-validated self-administered questionnaire to collect data from hospital and community pharmacists. Convenience sampling was used to recruit 411 pharmacists. Data were analyzed using SPSS v27.0, employing descriptive and inferential statistics, including binary regression analysis and Spearman’s correlation.

**Results:**

Of the 411 study participants, 54.5% were female, and 44.5% were between the ages of 20 and 30. Regarding work experience, 35.3% had 6 to 10 years of professional experience, while 32.4% of the participants reported familiarity with MTM. A majority of participants (69.3%, *n* = 285) demonstrated good knowledge about MTM, while 51.1% (*n* = 210) exhibited a positive attitude, and 52.8% (*n* = 217) reported engaging in good MTM practices. Additionally, 54.3% (*n* = 223) of pharmacists reported encountering multiple barriers to the implementation of MTM services, including a lack of support or collaboration from physicians (74.2%), limited access to essential patient information such as laboratory results, charts, and patient history (73.5%), and a lack of authority to initiate, monitor, or adjust therapy based on patient needs (73.5%).

**Conclusion:**

The findings of this study indicate that while pharmacists demonstrated adequate knowledge of MTM services, their attitudes and practices were found to be inadequate. This highlights an urgent need for targeted interventions, such as continuing pharmacy education, to further enhance their MTM-related knowledge, attitudes, and professional competencies. Furthermore, policymakers should recognize and expand the role of pharmacists in providing MTM services to ensure better therapeutic outcomes and improved patient care.

**Trial registration:**

Not applicable.

**Supplementary Information:**

The online version contains supplementary material available at 10.1186/s40780-025-00493-8.

## Introduction

Drug-related problems (DRPs) remain a significant public health concern globally [1]. This mainly includes inappropriate drug selection, ineffective drug therapy, dosing errors, duplications in therapy, adverse drug events, drug interactions, contraindications, inappropriate dosage, and poor adherence, all of which may lead to adverse outcomes [2]. Unresolved or potential DRPs may result in unnecessary outpatient visits, hospitalizations, and prolonged care, thus disrupting clinical therapy and exacerbating patients’ financial burdens [3, 4]. In the United States, Data from 2021 indicate that DRPs account for around 3–6% of hospital admissions, resulting in around 700,000 deaths annually and incurring costs of 130 billion dollars each year [1].

Medication therapy management (MTM) is a vital patient-centered service. It significantly reduces drug and treatment-related complications, enhances access to suitable therapies, and empowers patients to actively manage their medications, thereby improving therapeutic outcomes [4–6]. The practical delivery of MTM typically involves five key components: a comprehensive medication review (medication therapy review, MTR), development of a personal medication record (PMR), creation of a patient-centered action plan (medication-related action plan, MAP), pharmacist interventions or referrals as needed (intervention and referral), and consistent documentation and follow-up to monitor patient progress [7, 8].

Pharmacists, given their expertise in pharmacotherapy and accessibility, play a crucial role in MTM by assisting patients in optimizing their treatment regimens [9–14]. They educate patients about medications and proper administration techniques, minimize adherence challenges, identify and resolve numerous medication-related issues, including drug-drug and drug-disease interactions, and monitor therapy adjustments in cases of renal and hepatic impairment [9–14]. Pharmacist-led MTM services have improved medication adherence, enhanced clinical and humanistic outcomes, reduced hospitalizations, decreased drug-related problems and lowered medication-related costs [15–19]. A growing body of evidence highlights the effectiveness and value of MTM, underscoring the critical importance of collaborative care between pharmacists, other healthcare providers, and patients in achieving better management of chronic conditions and reducing medication-related harm [20–22]. However, the success of MTM programs depends on several factors, including the knowledge, attitudes, and practices (KAP) of the pharmacists delivering these services [7]. For example, Adeoye et al. (2018) demonstrated that pharmacists’ attitudes significantly influence MTM completion rates [5], while Elayeh et al. (2017) emphasized that inadequate therapeutic knowledge and clinical problem-solving skills hinder the implementation of patient-focused services [23]. Therefore, assessing pharmacists’ knowledge and competencies is essential for anticipating challenges in MTM delivery and critical in program sustainability planning [6, 7]. KAP surveys offer valuable insights into pharmacists’ professional development needs, concerns, and challenges and serve as valuable tools for developing and evaluating effective public health interventions [24].

Despite the increasing global focus on MTM, there is a lack of research on pharmacists’ awareness, implementation, and engagement with MTM services within Pakistan’s community and hospital pharmacy settings. In contrast to other low- and middle-income countries (LMICs), such as Indonesia and Malaysia [4, 20], where governmental initiatives and established structured MTM programs are already in place, Pakistan persists in confronting distinct contextual challenges. The PharmD curriculum is predominantly theoretical, lacking a formal clerkship or apprenticeship, resulting in graduates being inadequately equipped for clinical positions. The swift increase in pharmacy graduates, coupled with insufficient practice opportunities, ambiguous professional roles, a physician-centric healthcare culture, and low patient recognition of pharmacists’ contributions, has further hindered the implementation of MTM. At the systemic level, Pakistan lacks formal MTM frameworks, reimbursement models, and collaboration agreements, while inadequate enforcement of pharmacy practice regulations and a fragmented healthcare system obstruct the consistent integration of pharmacists’ educational services.

To the best of our knowledge, this is the first study in Pakistan to thoroughly evaluate the KAP of hospital and community pharmacists on MTM and perceived barriers to its implementation. This study also investigates socio-demographic variation (age, income, working hours) within the early stage of the MTM setting, providing insights into preparedness and barriers that have not been comprehensively reported in Pakistan. The study aims to fill these gaps by assessing educational needs, understanding current attitudes, and identifying practice-related barriers, critical steps toward improving pharmacists’ contributions to MTM and enhancing pharmaceutical care services in Pakistan. The findings of this research may inform targeted strategies to strengthen the implementation of MTM services and promote advancements in clinical pharmacy practice, patient counseling, and interprofessional collaboration across nationwide healthcare settings.

## Methodology

The present study was performed according to the STROBE (Strengthening the Reporting of Observational Studies in Epidemiology) checklist [25].

### Ethical consideration

This study received approval from the Research Ethics Committee at Riphah International University, Islamabad, Pakistan (Reference Number REC-RIPS/2025/16). All study participants provided informed consent before the commencement of the study, and subsequent study procedures adhered to the guidelines outlined in the Declaration of Helsinki [26].

### Study design and study setting

This observational cross-sectional study was conducted from May to August 2024 among community and hospital pharmacists in Pakistan’s twin cities, Islamabad and Rawalpindi, across both public and private pharmacy settings.

### Study population

The study population comprised registered hospitals and community pharmacists employed in licensed pharmacies. Inclusion criteria were male and female pharmacists with at least six months of professional experience who consented to participate in the study. Exclusion criteria included intern pharmacists, pharmacy technicians, pharmacists with less than six months of professional experience, and individuals who refused to participate.

### Sample size and sampling techniques

According to the Pharmacy Council of Pakistan, there were approximately 63,875 registered pharmacists in the country as of 2025 [27]. Based on this population, the required sample size was calculated using the Rao-Soft online sample size calculator [28]. The calculations were performed with a 5% margin of error, a 95% confidence interval, and a 50% non-response rate, resulting in a required study sample size of at least 382 participants. A convenience sampling technique was used to approach the 487 registered pharmacists who requested to participate in the study, of whom 411 completed the questionnaire, yielding a response rate of 84.4%.

### Development of questionnaire

The study questionnaire was developed based on an extensive review of relevant literature published on MTM and previously validated survey instruments [20, 29–32]. The study questionnaire had five sections. The first section collected pharmacists’ demographic and work-related information, such as gender, age, education, marital status, experience (years), practice city, practice setting, daily prescriptions, monthly income, daily working hours, and familiarity with MTM. Section 2 assessed pharmacists’ knowledge of MTM through ten true/false items addressing its core purpose, benefits, and impact on chronic disease management and healthcare costs. Section 3 evaluated their attitudes toward MTM, using a five-point Likert scale to capture pharmacists’ agreement with its usefulness for patients and the role of pharmacists in delivering these services. Section 4 measured MTM-related current practice by asking how frequently pharmacists engaged in MTM-related activities, such as assessing patients, developing treatment plans, reviewing medications, and communicating with other healthcare providers, and Sect. 5 identified the perceived barriers to implementing MTM services, covering challenges related to documentation, interprofessional collaboration, and organizational support.

Afterwards, a research committee of three academic experts in clinical pharmacy practice validated its content. These experts evaluated the questionnaire items for wording, clarity, and relevance to the study’s aims and objectives. Their feedback was reviewed and incorporated to refine the questionnaire. Face validity was verified by conducting a pilot study. A preliminary questionnaire was pilot-tested with 23 pharmacists to assess its clarity and comprehension. The feedback from this pilot test led to further modifications, ensuring the questionnaire was aligned with the study’s objectives. The participants from the pilot study were excluded from the final analysis. Finally, the reliability of the finalized questionnaire was assessed using Cronbach’s alpha in SPSS Version 27. The overall Cronbach’s alpha was 0.836, with subscale values of 0.888 for attitude, 0.885 for practice, and 0.813 for barriers. These values indicate that the questionnaire demonstrated good internal consistency, with Cronbach’s alpha values exceeding 0.70.

### Scoring of questionnaire

The knowledge section consisted of 10 true-or-false items, with each correct response earning one point, resulting in a maximum possible score of 10. The attitude section included six items measured on a 5-point Likert scale ranging from 1 (strongly disagree) to 5 (strongly agree), resulting in a maximum score of 30. The practice section consisted of eight items, also measured on a 5-point Likert scale, where 1 indicated “never,” and 5 indicated “at all times,” with a maximum score of 40. The barriers section consisted of 23 items, assessed using the same 5-point Likert scale as the attitude section, producing a total possible score of 115.

Total scores for each section, knowledge, attitude, practice, and barriers were calculated by summing individual item scores. Participants were then categorized into two groups based on the median score of each respective section. Those scoring equal to or above the median were classified as having high knowledge, a positive attitude, good practice, or experiencing more barriers. In contrast, those scoring below the median were categorized as having low knowledge, a negative attitude, poor practice, or encountering fewer barriers.

### Data collection procedure

Data were collected for four months, from May 2024 to August 2024, using a validated, structured, self-administered questionnaire. The researchers visited the hospitals and community pharmacies to meet with pharmacists and explained the study’s aims and objectives. Upon receiving verbal consent, participants were provided with the pre-validated self-administered questionnaires. Most participants completed the survey and returned the questionnaires on the spot, with the entire procedure having a mean duration of 15–30 min. Participants who could not finish the questionnaire promptly due to workload constraints requested that the researchers retrieve the completed forms later. In these circumstances, the questionnaires were collected at a mutually agreed-upon time.

### Data analysis

The data were entered and statistically analyzed using SPSS version 27 (IBM Corporation, Chicago, IL, USA). Descriptive statistics were applied to summarize participants’ demographic characteristics and responses across the various sections of the questionnaire. Chi-square tests were performed to examine differences in Knowledge, Attitudes, Practices, and perceived Barriers based on participants’ demographic characteristics. Multivariable binary logistic regression was conducted to identify the potential associations between binary dependent and independent variables, with results reported as odds ratios and a 95% confidence interval. Spearman correlation coefficient analyses were conducted to determine the relationships among the Knowledge, Attitudes, Practices, and Barriers domains. A *p*-value of less than 0.05 was considered significant.

## Results

### Socio-demographic characteristics of the study population

A total of 411 pharmacists participated in the study. Among them, 54.5% were female (*n* = 224), and most participants were between 20 and 30 years of age (44.5%). Most pharmacists, 86.1%, held a Pharm D degree, and 51.6% were single. Regarding professional experience, 35.3% have 6 to 10 years of work experience. Participants were almost equally distributed between Islamabad, 52.6% and Rawalpindi, 47.4%. The majority practiced in hospital pharmacy settings, 64.7%, while 35.3% worked in community pharmacies. In terms of monthly income, 40.1% reported earning between PKR 50,000 to 100,000. Regarding working hours, 35.0% reported working 9 h per day. When asked about familiarity with MTM services, 42.8% reported being somewhat familiar. A detailed summary of the participants’ socio-demographic and work-related characteristics is presented in Table 1.

**Table 1:** Socio-Demographic characteristics of pharmacists

Socio-Demographic characteristics	Categories	*n* (%)
**Gender**	Female	224 (54.5)
	Male	187 (45.5)
**Age**	20–30	183 (44.5)
	31–40	165 (40.1)
	41–50	37 (9.0)
	Above 50	26 (6.3)
**Qualification**	Pharm D	354 (86.1)
	MPhil	38 (9.2)
	PhD	19 (4.6)
**Marital Status**	Single	212 (51.6)
	Married	199 (48.4)
**Experience**	**<** 1	103 (25.1)
	1–5	128 (31.1)
	6–10	145 (35.3)
	> 10	35 (8.5)
**Practice City**	Islamabad	216 (52.6)
	Rawalpindi	195 (47.4)
**Practice Setting**	Community pharmacy	145 (35.3)
	Hospital pharmacy	266 (64.7)
**Daily prescriptions**	1–35	128 (31.1)
	36–70	143 (34.8)
	> 70	140 (34.1)
**Monthly income (PKR)**	< 50 K	153 (37.2)
	50–100 K	165 (40.1)
	101–150 K	61 (14.8)
	> 150 K	32 (7.8)
**Working hours per week**	8 h	124 (30.2)
	9 h	144 (35.0)
	> 9 h	143 (34.8)
**Familiarity with MTM**	Very familiar	133 (32.4)
	Somewhat familiar	176 (42.8)
	I am not familiar with it at all.	102 (24.8)

PKR: Pakistani Rupees

### Knowledge of medication therapy management

The pharmacists demonstrated a generally high level of knowledge regarding MTM services. The mean knowledge score was 8.1 ± 1.3, with a median score of 8 and an interquartile range (IQR) of 7–9. Based on the classification, 69.3% of the participants were categorized as having high knowledge, while 30.7% had low knowledge. Among the individual knowledge items, over 80% of pharmacists correctly identified that MTM services help achieve optimal therapeutic outcomes, involve individualized treatment plans, and play a key role in chronic disease management and cost reduction. The distribution of knowledge scores across individual statements and overall classification into high and low knowledge groups are provided in Supplementary Table 1.

### Attitudes toward medication therapy management

The participants’ attitudes toward MTM services were generally positive. The mean attitude score was 23.9 ± 5.1, with a median score of 25 (IQR: 22–27). Slightly over half (51.1%) of the pharmacists exhibited a positive attitude, while 48.9% showed a negative attitude. A large proportion of pharmacists agreed or strongly agreed that reviewing patients’ medication profiles and providing interventions are essential for pharmacists and that MTM services could enhance patient outcomes and expand pharmacists’ roles. The detailed breakdown of pharmacists’ attitudes toward MTM services, including item-wise responses and overall attitude classification, is shown in Supplementary Table 2.

### Practices related to medication therapy management

Regarding actual practice, the mean practice score was 28.4 ± 6.8, with a median score of 29 (IQR: 24–34). Slightly more than half of the pharmacists (52.8%) demonstrated good practice behaviors, while 47.2% were categorized as having poor practices. The most frequently performed MTM activities by pharmacists commonly include assessing patient health status, monitoring therapy responses, providing patient education, and supporting adherence. In contrast, formulating a medication treatment plan emerged as one of the least performed activities. Similarly, activities like initiating or modifying medication therapy were also reported less frequently. The pharmacists’ self-reported practices regarding MTM activities, along with overall practice scores, are outlined in Supplementary Table 3.

### Barriers to the implementation of MTM services

The pharmacists faced numerous barriers in implementing MTM services. The mean barrier score was 84.9 ± 10.8, with a median of 85 (IQR: 79–92). 54.3% of respondents faced more barriers, whereas 45.7% reported fewer barriers. The most commonly reported barriers included lack of time, limited access to patient information, lack of collaborative agreements with physicians, and insufficient support from management or leadership. Financial challenges, including a lack of reimbursement mechanisms, were also notable obstacles. The perceived barriers to MTM implementation, including responses to individual barrier items and the overall barrier classification, are summarized in Supplementary Table 4.

### Association of socio-demographic factors with knowledge, attitudes, practices, and barriers

Chi-square analysis revealed that age was significantly associated with practice (*p* = 0.015) and barriers (*p* = 0.013). Pharmacists aged 41–50 years exhibited better practice behaviors and reported facing fewer barriers compared to younger age groups. Similarly, monthly income showed a significant association with practice, whereas pharmacists earning higher income demonstrated better practice scores (*p* = 0.006). Pharmacists with longer working hours reported better practice behaviors (*p* = 0.048). Additionally, pharmacists who were more familiar with MTM services had a more positive attitude (*p* = 0.008) and reported facing fewer barriers (*p* = 0.000). Other variables such as gender, marital status, practice city, and practice setting did not show statistically significant associations with knowledge, attitude, practice, or barriers (*p* > 0.05). Table 2. Knowledge, attitude, practice, and barrier by sample characteristics.

**Table 2:** Knowledge, attitude, practice, and barrier by sample characteristics

Socio-Demographic characteristics	Knowledge			Attitude			Practice			Barrier		
	Low *n* (%)	High *n* (%)	χ2 (*P*)	Negative *n* (%)	Positive *n* (%)	χ2 (*P*)	Poor *n* (%)	Good *n* (%)	χ2 (*P*)	Fewer *n* (%)	More *n* (%)	χ2 (*P*)
**Total**	126 (30.7)	285 (69.3)		201 (48.9)	210 (51.1)		194 (47.2)	217 (52.8)		188 (45.7)	223 (54.3)	
**Gender**			0.81 (0.775)			0.812 (0.368)			0.717 (0.397)			0.495 (0.482)
Female	70 (31.3)	154 (68.8)		105 (46.9)	119 (53.1)		110 (49.1)	114 (50.9)		106 (47.3)	118 (52.7)	
Male	56 (29.9)	131 (70.1)		96 (51.3)	91 (48.7)		84 (44.9)	103 (55.1)		82 (43.9)	105 (56.1)	
**Age**			1.733 (0.630)			3.961 (0.266)			10.463 (**0.015**)			10.775 (**0.013**)
20–30	51 (27.9)	132 (72.1)		98 (53.6)	85 (46.4)		96 (52.5)	87 (47.5)		90 (49.2)	93 (50.8)	
31–40	53 (32.1)	112 (67.9)		71 (43.0)	94 (57.0)		75 (45.5)	90 (54.5)		78 (47.3)	87 (52.7)	
41–50	14 (37.8)	23 (62.2)		19 (51.4)	18 (48.6)		9 (24.3)	28 (75.7)		16 (43.2)	21 (56.8)	
Above 50	8 (30.8)	18 (69.2)		13 (50.0)	13 (50.0)		14 (53.8)	12 (46.2)		4 (15.4)	22 (84.6)	
**Qualification**			0.388 (0.824)			0.138 (0.933)			5.919 (0.052)			3.093 (0.213)
Pharm D	107 (30.2)	247 (69.8)		172 (48.6)	182 (51.4)		159 (44.9)	195 (55.1)		166 (46.9)	188 (53.1)	
MPhil	12 (31.6)	26 (68.4)		19 (50.0)	19 (50.0)		22 (57.9)	16 (42.1)		17 (44.7)	21 (55.3)	
PhD	7 (36.8)	12 (63.2)		10 (52.6)	9 (47.4)		13 (68.4)	6 (31.6)		5 (26.3)	14 (73.7)	
**Marital Status**			1.646 (0.200)			0.280 (0.597)			3.856 (0.050)			0.360 (0.549)
Single	59 (27.8)	153 (72.2)		101 (47.6)	111 (52.4)		110 (51.9)	102 (48.1)		110 (47.2)	112 (52.8)	
Married	67 (33.7)	132 (66.3)		100 (50.3)	99 (49.7)		84 (42.2)	115 (57.8)		88 (44.2)	111 (55.8)	
**Experience**			6.327 (0.097)			1.953 (0.582)			3.837 (0.280)			6.953 (0.073)
**<** 1	33 (32.0)	70 (68.0)		52 (52.4)	49 (47.6)		50 (48.5)	53 (51.5)		50 (48.5)	53 (51.5)	
1–5	29 (22.7)	99 (77.3)		60 (46.9)	68 (53.1)		64 (50.0)	64 (50.0)		57 (44.5)	71 (55.5)	
6–10	53 (36.6)	92 (63.4)		73 (50.3)	72 (49.7)		60 (41.4)	85 (58.6)		72 (49.7)	73 (50.3)	
> 10	11 (31.4)	24 (68.6)		14 (40.0)	21 (60.0)		20 (57.1)	15 (42.9)		9 (25.7)	26 (74.3)	
**Practice City**			0.226 (0.634)			0.516 (0.473)			0.962 (0.327)			0.402 (0.526)
Islamabad	64 (29.6)	152 (70.4)		102 (47.2)	114 (52.8)		97 (44.9)	119 (55.1)		102 (47.2)	114 (52.8)	
Rawalpindi	62 (31.8)	133 (68.2)		99 (50.8)	96 (49.2)		97 (49.7)	98 (50.3)		86 (44.1)	109 (55.9)	
**Practice Setting**			0.302 (0.583)			0.713 (0.399)			0.255 (0.613)			0.075 (0.783)
Community	42 (29.0)	103 (71.0)		75 (51.7)	70 (48.3)		66 (45.5)	79 (54.5)		65 (44.8)	80 (55.2)	
Hospital	84 (31.6)	182 (68.4)		126 (47.4)	140 (52.6)		128 (48.1)	138 (51.9)		123 (46.2)	143 (53.8)	
**Daily Prescriptions**			0.003 (0.998)			0.011 (0.994)			1.181 (0.554)			1.514 (0.469)
1–35	39 (30.5)	89 (69.5)		63 (49.2)	65 (50.8)		56 (43.8)	72 (56.3)		64 (50.0)	64 (50.0)	
36–70	44 (30.8)	99 (69.2)		70 (49.0)	73 (51.0)		72 (50.3)	71 (49.7)		61 (42.7)	82 (57.3)	
> 70	43 (30.7)	97 (69.3)		68 (48.6)	72 (51.4)		66 (47.1)	74 (52.9)		63 (45.0)	77 (55.0)	
**Monthly income** **(PKR)**			1.957 (0.581)			2.004 (0.572)			12.292 (**0.006**)			3.192 (0.363)
< 50 K	44 (28.8)	109 (71.2)		79 (51.6)	74 (48.4)		83 (54.2)	70 (45.8)		74 (48.4)	79 (51.6)	
50–100 K	49 (29.7)	116 (70.3)		77 (46.7)	88 (53.3)		71 (43.0)	94 (57.0)		79 (47.9)	86 (52.1)	
101–150 K	20 (32.8)	41 (67.2)		27 (44.3)	34 (55.7)		20 (32.8)	41 (67.2)		23 (37.7)	38 (62.3)	
> 150 K	13 (40.6)	19 (59.4)		18 (56.3)	14 (43.8)		20 (62.5)	12 (37.5)		12 (37.5)	20 (62.5)	
**Working hours per week**			3.400 (0.183)			0.999 (0.607)			6.060 (**0.048**)			4.042 (0.133)
8 h	35 (28.2)	89 (71.8)		56 (45.2)	68 (54.8)		49 (39.5)	75 (60.5)		66 (53.2)	58 (46.8)	
9 h	39 (27.1)	105 (72.9)		73 (50.7)	71 (49.3)		67 (46.5)	77 (53.5)		62 (43.1)	83 (56.9)	
> 9 h	52 (36.4)	91 (63.6)		72 (50.3)	71 (49.7)		78 (54.5)	65 (45.5)		60 (42.0)	83 (58.0)	
**Familiarity with MTM**			0.274 (0.872)			9.589 (**0.008**)			2.074 (0.354)			24.411 (**0.000**)
Very familiar	43 (32.3)	90 (67.7)		56 (42.1)	77 (57.9)		56 (42.1)	77 (57.9)		45 (33.8)	88 (66.2)	
Somewhat familiar	53 (30.1)	123 (69.9)		82 (46.6)	94 (53.4)		88 (50.0)	88 (50.0)		76 (43.2)	100 (56.8)	
I am not familiar with it at all.	30 (29.4)	72 (70.6)		63 (61.8)	39 (38.2)		50 (49.0)	52 (51.0)		67 (65.7)	35 (34.3)	

### Factors influencing knowledge, attitudes, practices, and barriers

The regression analysis showed no significant association between knowledge levels and socio-demographic characteristics, including gender, age, qualification, marital status, experience, practice setting, or familiarity with MTM (*p* > 0.05). However, familiarity with MTM emerged as a strong predictor of positive attitudes. Pharmacists who reported being “very familiar” with MTM services were more than twice as likely to have a positive attitude than those unfamiliar (OR = 2.17, 95% CI: 1.21–3.91, *p* = 0.009). Similarly, “somewhat familiar” also had higher odds of exhibiting a positive attitude (OR = 1.75, 95% CI: 1.02–3.02, *p* = 0.042). Pharmacist practice behaviors had a significant association with working hours and income levels. Pharmacists working 8 h per day were significantly more likely to demonstrate good practice behaviors than those working longer hours (OR = 2.05, 95% CI: 1.19–3.53, *p* = 0.009). Additionally, those earning a monthly income between 101,000 and 150,000 PKR were almost three times more likely to report good practices than their higher-earning counterparts (OR = 2.92, 95% CI: 1.07–7.96, *p* = 0.036). Moreover, pharmacist age and familiarity with MTM services were significantly associated with barriers faced. Regarding age, younger pharmacists aged 20–30 years and 31–40 years were significantly less likely to report facing more barriers than pharmacists over 50 (*p* = 0.010 and *p* = 0.008, respectively). Furthermore, pharmacists who were more familiar with MTM services reported fewer barriers. Those who were very familiar had over three times higher odds of facing fewer barriers compared to those not familiar (OR = 3.59, *p* = 0.001), and a similar trend was observed for those who were somewhat familiar (OR = 2.31, *p* = 0.003). Interestingly, a positive attitude toward MTM and good practice behaviors were both associated with a reduction in perceived barriers. Pharmacists with a positive attitude were less likely to report facing multiple barriers (OR = 0.62, 95% CI: 0.41–0.96, *p* = 0.034), and those demonstrating good practice were also less likely to encounter barriers (OR = 0.60, 95% CI: 0.38–0.93, *p* = 0.024). A detailed overview of the regression analysis findings is presented in Table 3.

**Table 3:** Regression analysis for factors associated with good knowledge, attitude, practice, and barriers regarding medication therapy management services

Socio-Demographic characteristics	Knowledge		Attitude		Practice		Barrier	
	OR (95% CI)	*P*-value	OR (95% CI)	*P*-value	OR (95% CI)	*P*-value	OR (95% CI)	*P*-value
**Gender**								
Female	0.94 (0.57–1.54)	0.818	1.03 (0.65–1.64)	0.884	0.88 (0.55–1.42)	0.627	0.89 (0.55–1.44)	0.642
Male	1.00	-	1.00	-	1.00	-	1.00	-
**Age**								
20–30	0.72 (0.21–2.41)	0.602	0.83 (0.27–2.57)	0.751	1.05 (0.33–3.36)	0.924	0.16 (0.04–0.64)	**0.010**
31–40	0.68 (0.24–1.94)	0.479	1.55 (0.57–4.17)	0.386	1.42 (0.51–3.94)	0.493	0.17 (0.50–0.64)	**0.008**
41–50	0.62 (0.19–2.04)	0.438	1.18 (0.38–3.71)	0.766	3.50 (1.02–12.03)	**0.046**	0.23 (0.05–0.94)	**0.041**
Above 50	1.00	-	1.00	-	1.00	-	1.00	-
**Qualification**								
Pharm D	0.91 (0.31–2.66)	0.867	1.57 (0.56–4.41)	0.387	2.62 (0.85–8.07)	0.093	0.37 (0.11–1.25)	0.112
MPhil	0.89 (0.25–3.20)	0.867	1.45 (0.43–4.88)	0.547	1.29 (0.35–4.76)	0.697	0.43 (0.10–1.71)	0.233
PhD	1.00	-	1.00	-	1.00	-	1.00	-
**Marital Status**								
Single	1.20 (0.72–1.98)	0.474	1.35 (0.84–2.18)	0.205	0.78 (0.48–1.26)	0.317	1.15 (0.71–1.87)	0.561
Married	1.00	-	1.00	-	1.00	-	1.00	-
**Experience**								
**<** 1	0.70 (0.21–2.29)	0.565	0.58 (0.18–1.82)	0.359	1.90 (0.61–5.87)	0.264	0.80 (0.24–2.69)	0.728
1–5	1.17 (0.40–3.46)	0.764	0.57 (0.20–1.61)	0.289	1.35 (0.48–3.80)	0.566	0.85 (0.27–2.60)	0.779
6–10	0.63 (0.24–1.62)	0.343	0.45 (0.17–1.15)	0.097	1.26 (0.49–3.21)	0.626	0.53 (0.19–1.49)	0.233
> 10	1.00	-	1.00	-	1.00	-	1.00	-
**Practice City**								
Islamabad	1.18 (0.76–1.84)	0.442	1.17 (0.77–1.77)	0.444	1.12 (0.73–1.70)	0.588	0.91 (0.59–1.40)	0.683
Rawalpindi	1.00	-	1.00	-	1.00	-	1.00	-
**Practice Setting**								
Community	1.27 (0.76–2.11)	0.358	0.98 (0.61–1.59)	0.958	1.08 (0.66–1.77)	0.749	1.22 (0.74–2.01)	0.427
Hospital	1.00	-	1.00	-	1.00	-	1.00	-
**Daily Prescriptions**								
1–35	1.07 (0.61–1.87)	0.797	1.14 (0.68–1.92)	0.607	1.36 (0.80–2.30)	0.254	1.03 (0.60–1.76)	0.900
36–70	1.05 (0.62–1.78)	0.848	1.04 (0.63–1.70)	0.868	0.86 (0.52–1.42)	0.566	1.21 (0.72–2.02)	0.466
> 70	1.00	-	1.00	-	1.00	-	1.00	-
**Monthly income** **(PKR)**								
< 50 K	1.58 (0.51–4.88)	0.424	1.60 (0.53–4.80)	0.395	1.31 (0.43–3.91)	0.628	1.57 (0.49–5.02)	0.447
50–100 K	1.77 (0.65–4.79)	0.256	2.20 (0.81–5.94)	0.117	2.08 (0.77–5.60)	0.146	1.70 (0.59–4.87)	0.323
101–150 K	1.61 (0.59–4.37)	0.349	1.91 (0.70–5.17)	0.200	2.92 (1.07–7.96)	**0.036**	1.64 (0.56–4.82)	0.364
> 150 K	1.00	-	1.00	-	1.00	-	1.00	-
**Working hours per week**								
8 h	1.52 (0.87–2.64)	0.138	1.38 (0.81–2.34)	0.231	2.05 (1.19–3.53)	**0.009**	0.64 (0.37–1.10)	0.110
9 h	1.62 (0.95–2.75)	0.072	0.97 (0.59–1.60)	0.918	1.45 (0.87–2.41)	0.149	1.07 (0.64–1.81)	0.774
> 9 h	1.00	-	1.00	-	1.00	-	1.00	-
**Familiarity with MTM**								
Very familiar	1.08 (0.57–2.03)	0.797	2.17 (1.21–3.91)	**0.009**	1.49 (0.81–2.73)	0.191	3.59 (1.95–6.59)	**0.000**
Somewhat familiar	1.06 (0.59–1.90)	0.840	1.75 (1.02–3.02)	**0.042**	1.06 (0.61–1.85)	0.818	2.31 (1.32–4.05)	**0.003**
I am not familiar with it at all.	1.00	-	1.00	-	1.00	-	1.00	-
**Knowledge**								
Low			1.10 (0.70–1.71)	0.667	1.04 (0.66–1.64)	0.848	1.28 (0.81–2.05)	0.283
High			1.00	-	1.00	-	1.00	-
**Attitude**								
Negative	1.09 (0.70–1.71)	0.679			1.07 (0.70–1.64)	0.739	0.62 (0.41–0.96)	**0.034**
Positive	1.00	-			1.00	-	1.00	-
**Practice**								
Poor	1.06 (0.67–1.68)	0.772	1.08 (0.70–1.65)	0.713			0.60 (0.38–0.93)	**0.024**
Good	1.00	-	1.00	-			1.00	-
**Barrier**								
Fewer	1.26 (0.80–2.01)	0.312	0.62 (0.40–0.95)	**0.030**	0.59 (0.38–0.93)	**0.022**		
More	1.00	-	1.00	-	1.00	-		

OR, odds ratio, CI, confidence interval, PKR: Pakistani Rupees

The *p*-values are bold where they are less than the significance level cut-off of 0.05

### Relationship between knowledge, attitudes, practices, and barriers

There was a significant negative correlation between knowledge and barriers (*r* = − 0.149, *p* = 0.002), indicating that pharmacists with higher knowledge tended to report fewer barriers to implementing MTM services. A positive correlation was observed between attitude and practice (*r* = 0.137, *p* = 0.006), indicating that more positive attitudes were associated with better engagement in MTM-related practices. Additionally, a significant positive correlation was found between attitude and perceived barriers (*r* = 0.352, *p* = 0.001), indicating that pharmacists who reported more barriers tended to maintain a relatively positive attitude toward MTM. Similarly, a weaker but significant positive correlation was also observed between practice and barriers (*r* = 0.127, *p* = 0.010), showing that those who engaged more actively in MTM practices also acknowledged the presence of particular challenges. Lastly, no statistically significant correlations were found between knowledge and attitude or knowledge and practice. The detailed correlation coefficients and significance values are summarized in Table 4.

**Table 4:** Spearman correlation between scores of knowledge, attitude, practice, and barrier

Variable	Correlation Coefficient	*P*-Value
Knowledge-Attitude	-0.077	0.118
Knowledge-Practice	0.041	0.409
Knowledge-Barrier	-0.149	**0.002**
Attitude-Practice	0.137	**0.006**
Attitude-Barrier	0.352	**0.000**
Practice-Barrier	0.127	**0.010**

* Correlation significant at 0.05 level (2-tailed)

## Discussion

As per the authors’ knowledge, this is the first study to provide a comprehensive assessment of hospital and community pharmacists’ KAP regarding MTM services and identify barriers to MTM implementation in Islamabad and Rawalpindi, Pakistan. Overall, pharmacists demonstrated high knowledge of MTM services, while their attitudes and practices were moderately positive. A substantial number of practice and policy-level barriers were reported. Furthermore, sociodemographic variables, particularly pharmacists’ age 41–50, those earning PKR 101k-150k, and those working standard 8-hour shifts, significantly influenced engagement with MTM services and their perception of barriers.

The Pakistani pharmacists exhibited good knowledge regarding MTM services, similar to findings from other LMICs [33, 34]. This suggests that knowledge is unlikely to hinder the provision of this extended service. This higher level of knowledge may be attributable to increased curricular exposure to clinical pharmacy, such as providing patient-centered services and research components, as well as supplementary training, or acquired through their experiential learning [35]. However, knowledge was weaker in areas such as herbal products, dietary supplements, and non-prescription MTM. Targeted continuing education and standardization of over-the-counter counselling practice might correct this shortcoming and enhance pharmacist proficiency.

Despite strong knowledge, pharmacists had only moderately positive attitudes, a trend also observed in other LMICs [6, 21, 36]. In high-income settings such as the United States [37] and Australia [38], favourable attitudes are supported by robust institutional integration, financial incentives, and legal recognition of MTM services. In contrast, in low-resource settings like Pakistan, where pharmacists recognize the potential of MTM services to enhance patient outcomes, but face systemic challenges and a lack of compensation that hamper their motivation to adopt their expanded roles. Walker et al. [39] found that unclear job roles, poor integration within the healthcare system, and professional marginalization can demotivate pharmacists from engaging in less visible yet essential responsibilities. These findings suggest that pharmacists’ attitudes are shaped not only by knowledge but also by the actual feasibility of role expansion in the existing healthcare environment.

Regarding practice, more than half of the pharmacists demonstrated good MTM practices, commonly performing basic activities such as therapy monitoring and patient assessment. However, fewer were reported to be involved in advanced critical activities, such as initiating or modifying medications. This trend aligns with findings from systematic reviews by Viswanathan et al. [40] and Marupuru et al., [18] where regulatory restrictions, workload pressures, or lack of administrative support and interprofessional collaboration impede full-scale MTM practice. A possible reason for such limited practice is that Pakistan’s healthcare system lacks formal MTM billing codes, limited acceptance of pharmacists’ expanded role by other healthcare professionals, the absence of national guidelines, or government-led MTM initiatives. Moreover, workload pressure and insufficient administrative assistance may further limit pharmacists’ ability to provide comprehensive MTM services. Studies from developed countries suggest that both the legal framework for pharmacists’ integration and reimbursement are essential for advanced MTM implementation [37, 38].

Furthermore, subgroup analysis revealed that older pharmacists (41–50 years) demonstrated significantly better practice and reported fewer barriers. This finding aligns with Alotaibi et al. [34], who found that MTM practices improve as experience increases. It is possible that more experienced pharmacists were better equipped to recognize and navigate systematic and institutional-level barriers, which may have allowed them to exercise greater confidence and autonomy, and to engage more effectively with physicians. In addition, pharmacists earning 101–150 K tend to adopt more favourable practices, similar to the trend observed in Qatar and Kuwait [41, 42]. One possible explanation is that pharmacists within this pay range may still be primarily involved in direct patient care, whereas those in the higher income brackets are more likely to assume administrative or managerial responsibilities, which reduce patient interaction and opportunities for MTM provision. Moreover, pharmacists working on a 9-hour standard shift had poorer practice than their counterparts. This difference may be related to a combination of factors, including burnout, insufficient staff, patient overload, and limited time for MTM service delivery. However, these interpretations remain speculative, and causality cannot be inferred from the present data. Future studies, ideally employing longitudinal or interventional designs, are needed to confirm these associations and explore underlying mechanisms.

In terms of barriers, pharmacists in our study reported a high number of barriers to MTM implementation, most commonly limited access to patient health records, lack of collaborative practice agreements, and inadequate financial incentives. Similar issues have been observed in Saudi Arabia, India, and Kuwait [34, 42, 43], even though these countries have better infrastructure than Pakistan. Policymakers should initiate a few necessary steps to overcome these barriers, such as digitalizing the documentation system, designing a multidisciplinary care model, and providing reimbursement mechanisms for pharmacists who offer MTM delivery.

Our findings also revealed that familiarity with MTM services was associated with both positive attitudes and fewer perceived barriers. Interestingly, pharmacists with lower MTM knowledge reported fewer barriers, possibly indicating an under recognition of the operational and systemic barriers to MTM delivery rather than the absence of such barriers. This is consistent with Shanableh et al. [44], who reported that early-career pharmacists underestimate structural limits until they acquire greater clinical exposure. Although education may initially enhance awareness of challenges, evidence from high-income settings shows that it ultimately equips pharmacists with the requisite skills, confidence, and collaborative networks to overcome these challenges [45, 46]. These observation supports the argument that MTM-focused training and certification can enhance patient care in low-resource settings, such as Pakistan. This aligns with the Theory of Planned Behaviour [47], which emphasizes that it is driven by knowledge, attitude, and perceived control collectively shaping behavioral intention. A pharmacist with better knowledge and resources can apply their expertise in MTM practices more effectively.

### Strengths and limitations

A notable strength of this study is that it is one of the first in Pakistan to comprehensively evaluate hospital and community pharmacists’ KAP regarding MTM services, helping to bridge a critical knowledge gap in the South Asian context. A validated and structured questionnaire was used to ensure the consistency and comparability of the collected data with international studies. Furthermore, the study examined the association between socio-demographic factors and pharmacists’ KAP toward MTM, as well as perceived barriers to its implementation. This facilitated a more thorough comprehension of subgroup disparities, allowing more precise recommendations for MTM in healthcare sectors. However, some limitations must be acknowledged. The cross-sectional design limits causal inferences, and the reliance on self-reported data introduces the possibility of reporting bias. Moreover, the study was geographically limited to Islamabad and Rawalpindi, which may not accurately reflect the diversity of experience and practice across Pakistan, especially in rural or underserved regions. Therefore, the findings should be interpreted with caution and not generalized to all pharmacists nationwide. Finally, the current study did not assess organizational or systemic-level factors (e.g., institutional policies, patient-related factors) due to resource constraints. Future qualitative research should be conducted to identify and understand these broader determinants of MTM implementation.

### Future implications

The findings emphasize the urgent need for structured training programs and Continuing Professional Development initiatives to enhance pharmacists’ understanding and confidence in delivering MTM services. Health authorities should consider formalizing collaborative practice agreements, developing standardized MTM protocols, and reimbursement mechanisms to support the sustainable integration of MTM into routine pharmacy practice. Incorporating MTM-related competences into the national pharmacy curricula and developing institution-specific regulations can further enhance the quality and longevity of service delivery.

Targeted interventions should be developed and tested to overcome the key barriers identified in this study, such as a lack of access to patient records, the absence of collaborative practice agreements, and time constraints. These may include introducing a digital documentation system, a multidisciplinary care model, or pilot reimbursement schemes specific to the local healthcare system. Future research should explore the perspectives of other stakeholders, including physicians, patients, and policymakers, to develop a multidimensional strategy for MTM adoption. Applying theoretical models such as the theory of planned behavior, the technology acceptance model, or the diffusion of innovations theory could provide a deeper understanding of the behavioral and systemic drivers of MTM implementation in Pakistan. Additionally, advanced statistical techniques such as path analysis would need to be performed in future research to examine the direct and indirect relationship between KAP and perceived barriers to MTM.

Further subgroup and stratified analyses are warranted to explore further the disparities in practice patterns across different age groups, experience levels, and income brackets. In addition, a qualitative studies among pharmacists could provide insight into workplace culture, clinical exposure, and access to mentorship. Finally, studies conducted in rural and underserved areas are needed to assess geographic variation among pharmacists’ KAP and to identify region-specific barriers to MTM service provision.

## Conclusion

As one of the first comprehensive KAP assessments on MTM in Pakistan, this study addresses a critical regional data gap and provides a foundation for nationwide research to further explore and expand the role of pharmacists in MTM services. The findings revealed that while pharmacists in Islamabad and Rawalpindi possess strong foundational knowledge about MTM services, their attitudes and actual practices are only moderately aligned with its principles. The implementation of MTM services is hindered by multiple systemic and institutional barriers, which are further influenced by socio-demographic factors. MTM practices can be advanced by addressing challenges through targeted policy interventions, such as infrastructural improvements, interprofessional collaboration, and educational reforms, including structured training and continuing medical education. The integration of pharmacists’ roles in MTM through legal frameworks and administrative support can lead to significant improvements in patient-centered care and reduce healthcare expenses.

## Supplementary Information

Below is the link to the electronic supplementary material.

Supplementary Material 1

Supplementary Material 2

Supplementary Material 3

## Data Availability

The datasets generated during and/or analyzed during the current study are available from the corresponding author upon reasonable request.
