# Supplementary material for: Medication therapy management in Pakistan: a cross-sectional evaluation of pharmacists’ knowledge, attitudes, practices, and barriers
Source: J Pharm Health Care Sci. 2025 Oct 6;11:85. doi: 10.1186/s40780-025-00493-8 (PMC12502235; doi:10.1186/s40780-025-00493-8)
Supplement: Supplementary file 1 — Supplementary Material 1 [file 40780_2025_493_MOESM1_ESM.docx]

**Medication Therapy Management in Pakistan: A Cross-Sectional Evaluation of Pharmacists’ Knowledge, Attitudes, Practices, and Barriers**

**Informed Consent**

Dear Pharmacist, We are conducting a research study to assess **pharmacists' knowledge, attitudes, and practices toward Medication Therapy Management (MTM) services** and explore the barriers to their implementation. We kindly invite you to participate by completing this brief **20-minute questionnaire**.

Your participation is voluntary, and all information you provide will be prote**cted**. The data collected will be used exclusively for **research purposes** and reported in aggregate form to ensure anonymity. There are no risks associated with participation, and you are free to withdraw from the study at any time without any consequence.

We greatly value your insights and contributions to this important research. Thank you for your time and participation.

**Participant's Signature: _______________ Date: ______________**

TOOL FOR DATA COLLECTION

Section 1. Demographic and work-related information

1. **Name (Optional):**
2. **Gender:** ☐ Female ☐ Male

**3. Age (Years):** ☐ 20-30 ☐ 31- 40 ☐ 41-50 ☐ Above 50

1. **Educational Qualification:** ☐ Pharm D ☐ MPhil ☐ PhD
2. **Marital status:** ☐ Single ☐ Married
3. **Experience (Years):** ☐< 1 ☐ 1-5 ☐ 6-10 ☐ More than 10
4. **Practice City:** ☐ Islamabad ☐ Rawalpindi
5. **Practice Setting:** ☐ Community Pharmacy ☐ Hospital Pharmacy
6. **Approximate (average) number of prescriptions handled by you daily:** ☐ 1-35 ☐ 36-70 ☐ >70

**10. Monthly income:** ☐ < 50K ☐ 50K-100K ☐ 101K-150K ☐ > 150k

1. **Your average working hours per week:** ☐ 8 hours ☐ 9 hours ☐ > 9 hours
2. **Rate your familiarity with Medication Therapy Management (MTM).**
   - Very familiar ☐ Somewhat familiar ☐ Not familiar at all

Section 2. Pharmacists' knowledge of medication therapy management

| **Statements** | **True** | **False** |
| --- | --- | --- |
| 1. **MTM is a service that helps patients achieve optimal therapeutic outcomes.** | ☐ | ☐ |
| 1. **MTM focuses on individualizing treatment plans for each patient.** | ☐ | ☐ |
| 1. **Core elements of MTM include the Medication Therapy Review (MTR), the Personal Medication Record (PMR), and the Medication-Related Action Plan (MAP).** | ☐ | ☐ |
| 1. **MTM aims to improve medication use, adherence, and patient understanding.** | ☐ | ☐ |
| 1. **MTM services help detect, prevent, and resolve medication-related problems.** | ☐ | ☐ |
| 1. **MTM services benefit patients using both prescription and non-prescription medications.** | ☐ | ☐ |
| 1. **MTM services are also helpful for patients taking herbal products or dietary supplements.** | ☐ | ☐ |
| 1. **A primary goal of MTM is to improve medication adherence.** | ☐ | ☐ |
| 1. **MTM plays an essential role in disease state management for chronic conditions.** | ☐ | ☐ |
| 1. **MTM helps decrease the total healthcare costs associated with chronic diseases.** | ☐ | ☐ |

**Section 3. Pharmacist's attitudes toward medication therapy management**

| **Statements** | **Strongly Disagree** | **Disagree** | **Neutral** | **Agree** | **Strongly Agree** |
| --- | --- | --- | --- | --- | --- |
| 1. **Besides normal dispensing functions, reviewing the patient's medication profile and providing interventions are essential roles of pharmacists to prevent adverse effects.** | ☐ | ☐ | ☐ | ☐ | ☐ |
| 1. **Patients would receive adequate and beneficial information from their providers about their chronic disease(s) and medication therapies by applying the MTM service.** | ☐ | ☐ | ☐ | ☐ | ☐ |
| 1. **Considering the core elements of MTM service, do you agree that MTM service is valuable?** | ☐ | ☐ | ☐ | ☐ | ☐ |
| 1. **Patients' health outcomes would be improved when pharmacists monitor medications compared to other healthcare providers.** | ☐ | ☐ | ☐ | ☐ | ☐ |
| 1. **Applying for MTM service requires more knowledge than elementary information about pharmacy practice.** | ☐ | ☐ | ☐ | ☐ | ☐ |
| 1. **Providing MTM service is a unique opportunity for pharmacists to participate in patient care on a broader spectrum.** | ☐ | ☐ | ☐ | ☐ | ☐ |

Section 4. Pharmacist's practices of medication therapy management services

| Statements | Never | Rarely | Sometimes | Most of the time | All times |
| --- | --- | --- | --- | --- | --- |
| 1. I am performing or obtaining necessary assessments of the patient's health status. | ☐ | ☐ | ☐ | ☐ | ☐ |
| 1. We are formulating a medication treatment plan. | ☐ | ☐ | ☐ | ☐ | ☐ |
| 1. I am selecting, initiating, modifying, or administering medication therapy. | ☐ | ☐ | ☐ | ☐ | ☐ |
| 1. We monitor and evaluate the patient's response to therapy, including safety and effectiveness. | ☐ | ☐ | ☐ | ☐ | ☐ |
| 1. Performing a comprehensive medication review to identify, resolve, and prevent medication-related problems, including adverse drug events. | ☐ | ☐ | ☐ | ☐ | ☐ |
| 1. We document the care delivered and communicate essential information to the patient's other primary care providers. | ☐ | ☐ | ☐ | ☐ | ☐ |
| 1. **We provide verbal education and training designed to enhance patient understanding and appropriate use of their medications.** | ☐ | ☐ | ☐ | ☐ | ☐ |
| 1. We provide information, support services, and resources to enhance patient adherence to their therapeutic regimens. | ☐ | ☐ | ☐ | ☐ | ☐ |

Section 5. Barriers to providing and implementing medication therapy management services

| Statements | Strongly Disagree | Disagree | Neutral | Agree | Strongly Agree |
| --- | --- | --- | --- | --- | --- |
| MTM Knowledge and Standards | | | | | |
| 1. Lack of clearly defined practice standards for MTM services | ☐ | ☐ | ☐ | ☐ | ☐ |
| 1. Lack of understanding of the components and goals of MTM services | ☐ | ☐ | ☐ | ☐ | ☐ |
| 1. Lack of awareness or availability of adequate educational MTM resources | ☐ | ☐ | ☐ | ☐ | ☐ |
| **Pharmacist Confidence and Capacity** | | | | | |
| 1. Lack of time to provide MTM services | ☐ | ☐ | ☐ | ☐ | ☐ |
| 1. Lack of training in therapeutic knowledge and clinical decision-making | ☐ | ☐ | ☐ | ☐ | ☐ |
| 1. Concerns about legal or professional consequences when providing MTM services | ☐ | ☐ | ☐ | ☐ | ☐ |
| Interprofessional Collaboration | | | | | |
| 1. Lack of formal recognition of pharmacists as healthcare providers | ☐ | ☐ | ☐ | ☐ | ☐ |
| 1. Limited access to essential patient information (e.g., labs, charts, medical history) | ☐ | ☐ | ☐ | ☐ | ☐ |
| 1. Absence of Collaborative Practice Agreements (CPAs) in the practice setting | ☐ | ☐ | ☐ | ☐ | ☐ |
| 1. Lack of support or collaboration from physicians or consultants | ☐ | ☐ | ☐ | ☐ | ☐ |
| 1. Limited trusted professional relationships with other healthcare providers | ☐ | ☐ | ☐ | ☐ | ☐ |
| Patient-Related Barriers | | | | | |
| 1. Lack of authority to initiate, monitor, or adjust therapy based on patient needs | ☐ | ☐ | ☐ | ☐ | ☐ |
| 1. Lack of patient willingness to engage in MTM services | ☐ | ☐ | ☐ | ☐ | ☐ |
| 1. Difficulty delivering MTM services due to cultural, language, or literacy barriers | ☐ | ☐ | ☐ | ☐ | ☐ |
| 1. Limited training or confidence in building patient relationships | ☐ | ☐ | ☐ | ☐ | ☐ |
| Organizational and Operational Barriers | | | | | |
| 1. Inadequate staffing (pharmacists, technicians, or support personnel) | ☐ | ☐ | ☐ | ☐ | ☐ |
| 1. Lack of ability or resources to market MTM services | ☐ | ☐ | ☐ | ☐ | ☐ |
| 1. Inadequate physical space or facilities for MTM service delivery | ☐ | ☐ | ☐ | ☐ | ☐ |
| 1. Lack of support from management or leadership | ☐ | ☐ | ☐ | ☐ | ☐ |
| Documentation Challenges | | | | | |
| 1. Absence of efficient and standardized documentation systems | ☐ | ☐ | ☐ | ☐ | ☐ |
| 1. Lack of time to properly document MTM interventions | ☐ | ☐ | ☐ | ☐ | ☐ |
| Financial Barriers | | | | | |
| 1. Limited financial resources to implement MTM services | ☐ | ☐ | ☐ | ☐ | ☐ |
| 1. Inability to obtain compensation or reimbursement for MTM services | ☐ | ☐ | ☐ | ☐ | ☐ |

Thank You
