# Supplementary material for: Medication therapy management in Pakistan: a cross-sectional evaluation of pharmacists’ knowledge, attitudes, practices, and barriers
Source: J Pharm Health Care Sci. 2025 Oct 6;11:85. doi: 10.1186/s40780-025-00493-8 (PMC12502235; doi:10.1186/s40780-025-00493-8)
Supplement: Supplementary file 2 — Supplementary Material 2 [file 40780_2025_493_MOESM2_ESM.docx]

**Table 1. Pharmacists' knowledge of medication therapy management**

| **Statement** | | **True**  **n (%)** | **False**  **n (%)** |
| --- | --- | --- | --- |
| **MTM is a service that helps patients achieve optimal therapeutic outcomes.** | | 335 (81.5) | 76 (18.5) |
| **MTM focuses on individualizing treatment plans for each patient.** | | 327 (79.6) | 84 (20.4) |
| **Core elements of MTM include the Medication Therapy Review (MTR), the Personal Medication Record (PMR), and the Medication-Related Action Plan (MAP).** | | 343 (83.5) | 68 (16.5) |
| **MTM aims to improve medication use, adherence, and patient understanding.** | | 327 (79.6) | 84 (20.4) |
| **MTM services help detect, prevent, and resolve medication-related problems.** | | 315 (76.6) | 96 (23.4) |
| **MTM services benefit patients using both prescription and non-prescription medications.** | | 346 (84.2) | 65 (15.8) |
| **MTM services are also helpful for patients taking herbal products or dietary supplements.** | | 328 (79.8) | 83 (20.2) |
| **A primary goal of MTM is to improve medication adherence.** | | 341 (83.0) | 70 (17.0) |
| **MTM plays an essential role in disease state management for chronic conditions.** | | 335 (81.5) | 76 (18.5) |
| **MTM helps decrease the total healthcare costs associated with chronic diseases.** | | 333 (81.0) | 78 (19.0) |
| **Overall Knowledge** | **Mean ± SD**  **Median (IQR)** | 8.1 ± 1.3  8 (7-9) | |
| **Knowledge Classification** | **Low Knowledge (0-7)**  **High Knowledge (8-10)** | 126 (30.7)  285 (69.3) | |

**Table 2. Pharmacists' attitudes toward medication therapy management**

| **Statements** | **Strongly Disagree**  **n (%)** | **Disagree**  **n (%)** | **Neutral**  **n (%)** | **Agree**  **n (%)** | **Strongly Agree**  **n (%)** | **Median (IQR)** |
| --- | --- | --- | --- | --- | --- | --- |
| **Besides the processes of normal dispensing functions, reviewing the patient's medication profile and providing interventions are essential roles of pharmacists to prevent adverse effects.** | 34 (8.3) | 12 (2.9) | 49 (11.9) | 143 (34.8) | 173 (42.1) | 4 (4-5) |
| **By applying the medication therapy management service, patients would receive adequate and beneficial information about their chronic disease(s) and medication therapies from their providers.** | 21 (5.1) | 13 (3.2) | 54 (13.1) | 187 (45.5) | 136 (33.1) | 4 (4-5) |
| **Considering the core elements of medication therapy management service, do you agree that its service is valuable?** | 18 (4.4) | 12 (2.9) | 56 (13.6) | 176 (42.8) | 149 (36.3) | 4 (4-5) |
| **Patients' health outcomes would be improved when pharmacists monitor medications compared to other healthcare providers.** | 19 (4.6) | 17 (4.1) | 52 (12.7) | 177 (43.1) | 146 (35.5) | 4 (4-5) |
| **Applying medication therapy management services requires more knowledge than elementary information about pharmacy practice.** | 17 (4.1) | 12 (2.9) | 59 (14.4) | 167 (40.6) | 156 (38.0) | 4 (4-5) |
| **Providing medication therapy management service is a unique opportunity for pharmacists to participate in patient care on a broader spectrum.** | 16 (3.9) | 33 (8.0) | 73 (17.8) | 160 (38.9) | 129 (31.4) | 4 (3-5) |
| **Overall Attitude** | **Mean ± SD**  **Median (IQR)** | | | 23.9 ± 5.1  25 (22-27) | | |
| **Attitude Classification** | **Negative Attitude (1-24)**  **Positive Attitude (25-30)** | | | 201 (48.9)  210 (51.1) | | |

**Table 3. Pharmacist's practices of medication therapy management services**

| **Statements** | **Never**  **n (%)** | **Rarely**  **n (%)** | **Sometimes**  **n (%)** | **Most of**  **Times**  **n (%)** | **All Times**  **n (%)** | **Median (IQR)** |
| --- | --- | --- | --- | --- | --- | --- |
| **I am performing or obtaining necessary assessments of the patient's health status.** | 20 (4.9) | 62 (15.1) | 78 (19.0) | 172 (41.8) | 79 (19.2) | 4 (3-4) |
| **We are formulating a medication treatment plan.** | 42 (10.2) | 85 (20.7) | 64 (15.6) | 146 (35.5) | 74 (18.0) | 4 (2-4) |
| **I am selecting, initiating, modifying, or administering medication therapy.** | 35 (8.5) | 83 (20.2) | 76 (18.5) | 149 (36.3) | 68 (16.5) | 4 (2-4) |
| **We monitor and evaluate the patient's response to therapy, including safety and effectiveness.** | 32 (7.8) | 62 (15.1) | 70 (17.0) | 148 (36.0) | 99 (24.1) | 4 (3-4) |
| **Performing a comprehensive medication review to identify, resolve, and prevent medication-related problems, including adverse drug events.** | 16 (3.9) | 59 (14.4) | 77 (18.7) | 164 (39.9) | 95 (23.1) | 4 (3-4) |
| **We document the care delivered and communicate**  **essential information to the patient's other primary care providers** | 31 (7.5) | 49 (11.9) | 70 (17.0) | 167 (40.6) | 94 (22.9) | 4 (3-4) |
| **We provide verbal education and training designed to enhance patient understanding and appropriate use of their medications.** | 12 (2.9) | 47 (11.4) | 62 (15.1) | 178 (43.3) | 112 (27.3) | 4 (3-5) |
| **We provide information, support services, and resources to enhance patient adherence to their therapeutic regimens.** | 17 (4.1) | 47 (11.4) | 65 (15.8) | 180 (43.8) | 102 (24.8) | 4 (3-4) |
| **Overall Practice** | **Mean ± SD**  **Median (IQR)** | | | 28.4 ± 6.8  29 (24-34) | | |
| **Practice Classification** | **Poor Practice (1-28)**  **Good Practice (29-40)** | | | 194 (47.2)  217 (52.8) | | |

**Table 4. Barriers to providing and implementing medication therapy management services**

| **Statements** | **Strongly Disagree**  **n (%)** | **Disagree**  **n (%)** | **Neutral**  **n (%)** | **Agree**  **n (%)** | **Strongly Agree**  **n (%)** | **Median (IQR)** |
| --- | --- | --- | --- | --- | --- | --- |
| **Lack of clearly defined practice standards for MTM services** | 14 (3.4) | 42 (10.2) | 78 (19.0) | 158 (38.4) | 119 (29.0) | 4 (3-5) |
| **Lack of understanding of the components and goals of MTM services** | 24 (5.8) | 26 (6.3) | 84 (20.4) | 153 (37.2) | 124 (30.2) | 4 (3-5) |
| **Lack of awareness or availability of adequate educational MTM resources** | 28 (6.8) | 32 (7.8) | 76 (18.5) | 125 (30.4) | 150 (36.5) | 4 (3-5) |
| **Lack of time to provide MTM services** | 26 (6.3) | 43 (10.5) | 72 (17.5) | 140 (34.1) | 130 (31.6) | 4 (3-5) |
| **Lack of training in therapeutic knowledge and clinical decision-making** | 15 (3.6) | 51 (12.4) | 76 (18.5) | 147 (35.8) | 122 (29.7) | 4 (3-5) |
| **Concerns about legal or professional consequences when providing MTM services** | 16 (3.9) | 35 (8.5) | 78 (19.0) | 162 (39.4) | 120 (29.2) | 4 (3-5) |
| **Lack of formal recognition of pharmacists as healthcare providers** | 15 (3.6) | 45 (10.9) | 69 (16.8) | 137 (33.3) | 145 (35.3) | 4 (3-5) |
| **Limited access to essential patient information (e.g., labs, charts, medical history)** | 27 (6.6) | 15 (3.6) | 67 (16.3) | 194 (47.2) | 108 (26.3) | 4 (3-5) |
| **Absence of Collaborative Practice Agreements (CPAs) in the practice setting** | 19 (4.6) | 39 (9.5) | 53 (12.9) | 182 (44.3) | 118 (28.7) | 4 (3-5) |
| **Lack of support or collaboration from physicians or consultants** | 11 (2.7) | 16 (3.9) | 79 (19.2) | 200 (48.7) | 105 (25.5) | 4 (3-5) |
| **Limited trusted professional relationships with other healthcare providers** | 18 (4.4) | 28 (6.8) | 88 (21.4) | 169 (41.1) | 108 (26.3) | 4 (3-5) |
| **Lack of authority to initiate, monitor, or adjust therapy based on patient needs** | 11 (2.7) | 26 (6.3) | 73 (17.8) | 166 (40.4) | 135 (32.8) | 4 (3-5) |
| **Lack of patient willingness to engage in MTM services** | 18 (4.4) | 31 (7.5) | 71 (17.3) | 174 (42.3) | 117 (28.5) | 4 (3-5) |
| **Difficulty delivering MTM services due to cultural, language, or literacy barriers** | 16 (3.9) | 23 (5.6) | 78 (19.0) | 189 (46.0) | 105 (25.5) | 4 (3-5) |
| **Limited training or confidence in building patient relationships** | 14 (3.4) | 26 (6.3) | 87 (21.2) | 187 (45.5) | 97 (23.6) | 4 (3-4) |
| **Inadequate staffing (pharmacists, technicians, or support personnel)** | 30 (7.3) | 55 (13.4) | 96 (23.4) | 163 (39.7) | 67 (16.3) | 4 (3-4) |
| **Lack of ability to market MTM services** | 25 (6.1) | 59 (14.4) | 85 (20.7) | 163 (39.7) | 79 (19.2) | 4 (3-4) |
| **Inadequate physical space or facilities for MTM service delivery** | 33 (8.0) | 61 (14.8) | 89 (21.7) | 128 (31.1) | 100 (24.3) | 4 (3-4) |
| **Lack of support from management or leadership** | 33 (8.0) | 68 (16.5) | 110 (26.8) | 138 (33.6) | 62 (15.1) | 3 (3-4) |
| **Absence of efficient and standardized documentation systems** | 33 (8.0) | 79 (19.2) | 83 (20.2) | 141 (34.3) | 75 (18.2) | 4 (2-4) |
| **Lack of time to properly document MTM interventions** | 32 (7.8) | 83 (20.2) | 111 (27.0) | 119 (29.0) | 66 (16.1) | 3 (2-4) |
| **Limited financial resources to implement MTM services** | 22 (5.4) | 49 (11.9) | 95 (23.1) | 168 (40.9) | 77 (18.7) | 4 (3-4) |
| **Inability to obtain compensation or reimbursement for MTM services** | 22 (5.4) | 50 (12.2) | 81 (19.7) | 182 (44.3) | 76 (18.5) | 4 (3-4) |
| **Overall Barrier** | **Mean ± SD**  **Median (IQR)** | | | 84.9 ± 10.8  85 (79-92) | | |
| **Barrier Classification** | **Fewer Barrier Faced (1-84)**  **More Barriers Faced** | | | 188 (45.7)  223 (54.3) | | |
